# Commentary on two recently published formal guidelines on management of “mosaic” embryos after preimplantation genetic testing for aneuploidy (PGT-A)

**DOI:** 10.1186/s12958-021-00716-1

**Published:** 2021-02-18

**Authors:** Norbert Gleicher, David H. Barad, Zion Ben-Rafael, Demian Glujovsky, Lyka Mochizuki, Deepak Modi, Maximillian Murtinger, Pasquale Patrizio, Raoul Orvieto, Shizuko Takahashi, Andrea Weghofer, Søren Ziebe, on behalf of the International Do No Harm Group in IVF (IDNHG-IVF)

**Affiliations:** 1 The Center for Human Reproduction, 21 East 69th Street, New York, NY 10021 USA; 2 The Foundation for Reproductive Medicine, New York, NY USA; 3 Stem Cell Biology and Molecular Embryology Laboratory, The Rockefeller University, New York, NY USA; 4 Department of Obstetrics and Gynecology, Medical University of Vienna, Vienna, Austria; 5 Department of Obstetrics and Gynecology and IVF, Laniado Medical Center, Netanya, Israel; 6 Fertility Argentina, Buenos Aires, Argentina; 7 Molecular and Cellular Biology Laboratory, ICRM-National Institute for Research in Reproductive Health, Mumbai, India; 8 NextXlinic IVF Centers Prof Zech, Bregenz, Austria; 9 Yale Fertility Center, Department of Obstetrics, Gynecology and Reproductive Sciences, Division of Reproductive Endocrinology and Infertility, Yale University, New Haven, CT USA; 10 Infertility and IVF Unit, Department of Obstetrics and Gynecology, Sheba Medical Center, Ramat Gan and Sackler Faculty of Medicine, Tel-Aviv University, Tel-Aviv, Israel; 11 Department of Biomedical Ethics and the Department of Obstetrics and Gynecology, The University of Tokyo, Graduate School of Medicine, Tokyo, Japan; 12 The Fertility Clinic, Ringhospital, University Hospital of Copenhagen, Copenhagen, Denmark

**Keywords:** Preimplantation genetic testing for aneuploidy (PGT-A), In vitro fertilization (IVF), Mosaicism, Guidelines, Professional organizations

## Abstract

Two professional societies recently published opinions on the clinical management of “mosaic” results from preimplantation genetic testing for aneuploidy (PGT-A) in human blastocyst-stage embryos in associations with in vitro fertilization (IVF). We here point out three principal shortcomings: (i) Though a most recent societal opinion states that it should not be understood as an endorsement of the use of PGT-A, any discussion of how PGT-A should be clinically interpreted for all practical purposes does offer such an endorsement. (ii) The same guideline derived much of its opinion from a preceding guidance in favor of utilization of PGT-A that did not follow even minimal professional requirements for establishment of practice guidelines. (iii) Published guidelines on so-called “mosaic” embryos from both societies contradict basic biological characteristics of human preimplantation-stage embryos. They, furthermore, are clinically unvalidated and interpret results of a test, increasingly seen as harmful to IVF outcomes for many infertile women. Qualified professional organizations, therefore, should finally offer transparent guidelines about the utilization of PGT-A in association with IVF in general.

This is a commentary of the International Do No Harm Group in IVF (IDNHG-IVF) on recently published practice guidelines issued by two professional societies on only one specific aspect of what is now called preimplantation genetic testing for aneuploidy (PGT-A), − the management of so-called “mosaic” embryos. We previously commented in detail on a guideline issued by the Preimplantation Genetic Diagnosis International Society (PGDIS) on the subject [1] and, therefore, will try not being repetitive. The IDNHG-IVF is a spontaneously coalesced group of clinicians, embryologists, biologists, as well as other scientists from all over the world associated with the practice of IVF who are concerned about the unvalidated addition of add-on practices to IVF, especially since 2010. These add-ons have been associated with declining live birth rates after IVF around the world, with PGT-A, likely, being the most consequential cause [1]. A more recent joint statement of the Practice Committee of the American Society for Reproductive Medicine (ASRM) and the society’s Genetic Counseling Professional Group (GCPG [2] (mostly made up of members of the PGDIS) is, however, a profoundly more important opinion in the fertility field. That this ASRM guidance in many aspects then relied on earlier, often incorrect, PGDIS representations is disappointing.

We welcome that the ASRM statement specifically pointed out that it “*does not endorse, nor does it suggest that PGT-A is appropriate in all cases of in vitro fertilization.*” At the same time, we, however, must conclude that offering advice on management of results of a test does constitute an endorsement of this test in the minds of most readers. This impression is further strengthened by the ASRM’s document not including increasingly serious concerns expressed in the field about PGT-A being an unvalidated test, for many patients offering no real benefit, and for at least some harmful in adversely affecting IVF outcomes [2, 3].

The subject here is the over 20 years-old hypothesis that determining whether an embryo is euploid or aneuploid before embryo transfer, will beneficially affect in vitro fertilization (IVF) outcomes by excluding chromosomal-abnormal embryos from transfer, thereby improving implantation, pregnancy and live birth chances of remaining embryos. In over 20 years, and in at least three different generations of the procedure now given the acronym PGT-A, investigators have, however, been unable to confirm the hypothesis either experimentally and/or in clinical practice. To the contrary, increasing evidence has been developed to suggest that, because of biological characteristics of human preimplantation-stage embryos, the hypothesis, simply, cannot work. Why that is, has been recently in detail reviewed [3] and, therefore, will here not be repeated. Consequently, the utility of PGT-A in clinical IVF practice is continuously challenged [4, 5]. Simply stated, if something does not work in over 20 years, why are we continuing to use it?

This is, indeed, the first question that must be asked, − and not whether ASRM specifically *“endorses”* PGT-A or whether PGT-A “*is appropriate for all cases of IVF”* (as the document states). The immediate question to follow is, however, why does ASRM at this point issues a policy statement on how to manage “mosaic” embryos after diagnosis by PGT-A in the first place? Would not a statement be more appropriate that, finally, after over 20 years of failed attempts in confirming the PGT-A hypothesis, acknowledges that PGT-A simply, no longer should be used in routine clinical IVF practice?

By discussing the result of a test and claiming to offer advice on how clinicians should interpret it, an impression of endorsement cannot be avoided. Why, otherwise, bother, when so many important issues in infertility are awaiting formal guidance from qualified professional organizations? Interestingly, the American College of Obstetricians and Gynecologists (ACOG) has so-far also chosen *not* to address this issue in a formal opinion.

In three formal statements about PGT-A and its predecessor formats between 2008 and 2020 [2, 6, 7], ASRM has not publicly acknowledged that (i) PGT-A was never clinically validated for any of its claims of clinical utility in each of its three itinerations; (ii) Like any other diagnostic test, PGT-A should never have been introduced to the market place without prior validation studies (appropriate RCTs); (iii) that because this tests determines whether human embryos are disposed of or not, special ethical considerations given to human embryos mandate higher scrutiny for PGT-A than for almost any other diagnostic test [8]; (iv) indisputable evidence as of this point demonstrates that PGT-A leads to non-use or disposal of significant numbers of embryos with at least normal pregnancy and live-birth potential [9]. (v) Even excluding from consideration disputes over the validity of many published clinical studies, it is difficult to conclude that PGT-A should be used outside of investigational frameworks [1, 3, 5].

## Why a correct definition of mosaicism is essential?

Appropriately, the ASRM document initiated the discussion of “mosaic” results in PGT-A with a definition of mosaicism. By appropriating, however, an incorrect definition of mosaicism, presented by the PGDIS in 2016 in a first guideline document [10], and ever since followed by PGT-A laboratories worldwide, the ASRM only reinforced the confusion surrounding this subject: The reason is that mosaicism is *not*, as PGDIS and now ASRM suggest, “*presence of more than one chromosomally distinct cell line in a single sample originating from one individual”* but, by international consensus, *“presence (anywhere) in an individual of normal and abnormal cells that are genotypically distinct and are derived from a single zygote”* [11] and this definition is not restricted to one, two or even more biopsy samples. The definition always involves a whole organism and, in this case, a complete embryo.

The difference between these two definitions lies at the very core of why PGT-A has become such a controversial procedure/test. As since 2016, the PGDIS’ first guidance, clinically applied, PGT-A does *not* determine whether an embryo is mosaic (i.e., exhibits anywhere within the embryo two or more unique cell lineages). PGT-A only determines whether a single random 5–6 cell biopsy of trophectoderm at blastocyst-stage contains two or more distinct cell lineages. A current diagnosis of mosaicism assigned to an embryo is, therefore, not only based on an incorrect definition of mosaicism but on the incorrect biological understanding what mosaicism at preimplantation stages really represents. As has been well documented [12], simply mathematically, a 5–6 cell trophectoderm biopsy cannot represent the complete embryo in all its cellular and chromosomal diversity.

To avoid confusion going forward, we, therefore, will in this manuscript use quotation marks when the term “mosaicism” is used with only reference to a single trophectoderm biopsy and will omit quotation marks when the term mosaicism is correctly used in representation of a complete blastocyst-stage embryo, at that stage containing an embryonic cell lineage (inner cell mass) and an extraembryonic cell lineage (trophectoderm), with the former creating the fetus and the latter the placenta. Incongruities between aneuploidy of trophectoderm biopsies and inner cell mass were recently again emphasized [13].

PGDIS guidelines since 2016 mandate Next Generation Sequencing (NGS) or similar technologies [10], since older diagnostic platform did not have the ability to detect more than one cell lineage in a biopsy specimen. To be able to diagnose more than one cell lineage is, however, of course a precondition for any diagnosis of mosaicism. To further understand the problems that can arise when “mosaicism” is misconstrued as mosaicism, it is important to review the three diagnostic possibilities a single trophectoderm biopsy can yield at blastocyst-stage:

(i) The trophectoderm biopsy contains only one euploid cell line.
  This embryo may be 100% euploid; it, however, also may be mosaic because it may have somewhere else in trophectoderm or inner cell mass one or more additional cell lineages. The only thing certain is that this embryo is not 100% aneuploid. It, however, may be fully euploid or mosaic.
(ii) The trophectoderm biopsy contains only one cell line that is 100% aneuploid.
  This embryo may be truly 100% aneuploid (likely meiotic) or may be mosaic because elsewhere in the embryo there may be euploid cells (in which case the biopsied aneuploid cells would likely be of mitotic origin).
(iii) The trophectoderm contains two (or more) cell lineages, one of which is euploid. Under current PGT-A reporting *only* this embryo is reported out as “mosaic.”
  This embryo is with certainty mosaic, though at which percentage it is euploid and aneuploid, cannot be reliably determined from a single trophectoderm biopsy because there, of course, may be aneuploid cells beyond the 5–6 cells obtained in a trophectoderm biopsy.

These three scenarios, therefore, clearly demonstrate that true embryo mosaicism may be present in an embryo independent of any current PGT-A result. Current PGT-A, however, only reports option (iii) as “mosaic” and, therefore, vastly underreports true mosaicism, while, at the same time, greatly overvaluing the clinical importance of the “mosaicism” PGT-A is reporting. This is also the reason why even prominent investigators still falsely claim that “mosaicism” in blastocyst-stage embryos represents only a rare phenomenon in low single numbers of embryos [14], while in vivo as well as in vitro single cell studies have conclusively demonstrated that mosaicism in blastocyst-stage embryos (and in artificially from stem cells produced human gastruloids) basically represents a normal physiological phenomenon in mouse models [15] and human embryos [16].

## Considerations regarding transfer of “mosaic’ embryos

As correctly noted in the recent ASRM document, a single trophectoderm biopsy will, likely, show a “mosaic” result in only 3–20% of cases [2]; with increasing biopsy numbers, percentages of detected “mosaicism” will, however, of course, increase. The ASRM document erred when stating that a single “mosaic” embryo biopsy may suggest a “*fully euploid embryo.*” This is, of course, technically impossible because presence of any second cell lineage in an organism fulfills the generally accepted definition of mosaicism [11] This, however, does not mean that the ASRM opinion erred in suggesting that transfer of “mosaic” embryos after PGT-A may result in a normal euploid pregnancies. That this can and is happening with similar outcomes to untested or PGT-A-tested embryos has been widely reported and has led by now to hundreds of healthy births [9]. These births, however, do not mean that the blastocyst-stage embryos from which these normal pregnancies arose were not mosaic at time of biopsy. As Bolton et al. in the mouse [15] and Yang et al. in humans demonstrated [16], embryos have a highly efficient ability to self-correct within the embryonic cell lineage downstream from blastocyst-stage by eliminating aneuploid cells through cell-death and apoptosis. Orvieto et al. recently in addition demonstrated that aneuploid embryos may self-correct downstream by expelling aneuploid cell fragments [17].

Following the same logic, the ASRM’s guidance is even more confusing when suggesting that “*an embryo with a ‘mosaic’ diagnosis may be fully aneuploid*” [2]. Considering the correct definition of mosaicism, this, too, is, of course, impossible: Even assuming only a minimal amount of normal-euploid DNA in an 5–6-cell trophectoderm biopsy that otherwise is aneuploid, such an embryo is, by definition, mosaic. Downstream evolution from euploid to aneuploid has, moreover, not been reported and appears highly unlikely.

Single cell investigations further suggested that ultimate embryo fate mostly depends on relative percentages of euploid and aneuploid cells within the embryonic cell lineage (i.e., inner cell mass). Self-correction is highly effective within the embryonic cell lineage up to approximately 50% aneuploidy. Even at higher aneuploidy percentages some embryos, however, still emerge as euploid newborns [15, 16]. Ploidy within the extraembryonic cell lineage (i.e., trophectoderm) appears of much less relevance in determining ultimate embryo fate, not the least because of discrepancies between trophectoderm and inner cell mass in percentages of aneuploid cells [13] and differences in ability to self-correct between the two cell lineages [15, 16].

The ASRM document correctly noted that “mosaic” blastocyst can, theoretically, give rise to a mosaic offspring, though also noted that such occurrences are apparently rare and, if they occur, clinically mostly irrelevant, though there is still a paucity of data on the subject [2]. What represents paucity is, of course, debatable: Already in early 2019, over 400 healthy births were reported worldwide after transfers of by PGT-A as “mosaic” or aneuploid tagged embryos [9]. Not even a single abnormal newborn with significant clinical consequences was delivered following such transfers. Numbers of delivered offspring must have significantly increased since, and we are, so-far, unaware of any newborn with significant handicap. Summarizing outcomes from transfer of “abnormal” embryos in the literature, the ASRM Opinion describes the so-far available experience of transferring chromosomal-abnormal embryos as, “*somewhat encouraging.*” We fully concur.

We, however, consider ASRM’s deferral to guidances from two other organizations surprising: the PGDIS [10] and the Congress on Controversies in Preconception, Preimplantation and Prenatal Genetic Diagnosis (CoGEN) (https://www.cogeneurope.eu/events/annual-conferences). Both groups and the publication partner of the ASRM in the recent document, the GCPG, [2] are closely intertwined in their respective memberships and are conflicted in advising on clinical utilization of PGT-A. This judgment is not made lightly and is, ultimately, based on the uncontested observation that none of these groups have followed what is considered standard criteria for establishment and reporting of practice guidelines [18].

Yet, especially the PGDIS, since 2016, has been the guiding force in determining worldwide PGT-A practice, first by publishing an unreferenced anonymous guidance without peer review on its website in 2016 [10], and, more recently in 2019, by publishing in a peer-reviewed journal yet another guidance on the transfer of mosaic embryos [19]. We previously addressed this document because of its significant scientific as well as procedural shortcomings [1], by experts described as typically flawed guideline processes [20]. The IDNHG-IVF, therefore, previously described the 2019 PGDIS guideline on “mosaic” PGT-A result interpretation as misleading [1], and initially was comforted by the ASRM announcement of an impending publication of independent guidance on the subject.

Though a significant improvement over the 2019 PGDIS document, the ASRM guidelines, nevertheless, were for several reasons disappointing: We were surprised by the uncritical acceptance and, indeed inclusion into the ASRM document, of clear misstatements from the 2016 and 2019 PGDIS guidelines, starting with the definition of “mosaicism.” Since we previously described our criticism of the 2019 PGDIS guidance [1], we here do not want to be repetitive. One crucially important issue, in the ASRM document only glossed over, must, however, be addressed: The PGDIS advises that, based on percentages of aneuploid DNA in a biopsy sample, “mosaicism” can be quantitated and that such quantitation correlates with pregnancy chances after transfer [10]. Inexplicitly, the ASRM guidance fully accepted this incorrect statement and, indeed, even included it into its recent guidance [2].

## The threshold concept

The ASRM document notes that, as proposed by the PGDIS, current PGT-A criteria offer a diagnosis of “mosaicism” based on detection of an intermediate chromosome copy numbers, between monosomy and disomy or disomy and trisomy on an NGS profile [2]. To quantitate embryo mosaicism, the PGDIS in 2016 added the so-called “threshold concept” to the diagnostic armamentarium of PGT-A [10], as just a few weeks earlier first suggested by Scott Jr. and Galliano [21]. This concept assumes that varying percentages of aneuploid DNA in a single trophectoderm biopsy have diagnostic as well as prognostic significance for IVF cycle outcomes. Both assumptions are, however, unsubstantiated and incorrect.

As noted earlier, the assumption that a single trophectoderm biopsy correctly reflects ploidy of the complete blastocyst-stage embryo is mathematically unsustainable [12]. Should a biopsy contain more than one cell lineage, it, furthermore, incorrectly assumes that NGS can accurately determine exact percentages of DNA for each cell lineage and that, whatever this percentage is, reflects the complete embryo and predicts its implantation, pregnancy and live birth potential [10, 22]. At least two studies have, however, quite categorically rejected such associations [23, 24], a finding that should not surprise, considering that percentages of DNA can never be accurately determined (see below for further detail) and that the degree of aneuploidy at blastocyst stage can downstream still significantly change through self-correction [15–17].

Yet, 2016 PGDIS guidelines fixed threshold of percentages of aneuploid DNA in defining euploid, “mosaic,” and aneuploid ranges of embryos, with 0–20% aneuploid DNA defining euploid embryos, 21–80% “mosaic” embryos,” and with 81–100% defining aneuploid embryos [10]. What these cut-offs were based on was initially left to imagination, except for the fact that 20% represented (and still does) the sensitivity threshold of NGS platforms in detecting a second cell lineage in a single biopsy specimen. Embryos currently under PGDIS guidelines signed out as normal-euploid, therefore, may very well be mosaic if a trophectoderm biopsy contains less than 20% aneuploid DNA.

Neither the 20% threshold between euploid and “mosaic”, nor the 80% cut-off between “mosaic” and aneuploid have any evidentiary basis in biology, have ever been tested experimentally and/or have ever been clinically validated. They were simply, as later acknowledged [25], based on biologically and technically incredibly naive assumptions, including: (i) Every trophectoderm biopsy contains five cells. (ii) These five cells, therefore, represent 100% of DNA; 1/5 aneuploid cells will, consequently, produce 20% aneuploid DNA in a single trophectoderm biopsy, 2/5 cells 40%, etc. (iii) 2–4 aneuploid cells, therefore, define “mosaicism,” while 5/5 cells define an aneuploid embryo.

These assumptions are, however, unsustainable: As any embryologist can attest to, it is simply impossible to determine how many intact cells a trophectoderm biopsy contains. It, therefore, is impossible to determine the denominator that defines 100% DNA. Even if cell number-dependent diagnostic criteria, however, were feasible and correct, accurate percentages can never really be determined because every embryo biopsy is a traumatic event, resulting in cell rupture and DNA spillage, both, of course, further potentially complicating establishment of percentages of aneuploid DNA. The 2016 PGDIS guidance on how to diagnose embryos in PGT-A [10], is, therefore, without realistic biological underpinnings.

The increasing confusion generated by PGDIS and now also ASRM guidelines is acknowledged even by proponents of PGT-A. A recently published opinion by Paulson and Treff [26] proposes that, *“because of questions of its clinical significance*,*”* the term “mosaicism” be abandoned in PGT-A reports and replaced by the phrase *“embryos with intermediate copy-numbers.”* The authors, thus, acknowledge the lack of purposes of the threshold concept, currently universally applied in PGT-A; their recommendation to change terminology, however, does nothing to the basic flaws of PGT-A which, even using this new diagnostic designation, still, remains without biological, mathematical or procedural underpinnings.

## Conclusions

The ASRM document also concluded that (regarding transfer of “mosaic” embryos), *“it may be premature to apply any for purposes of embryo-transfer decisions or for providing clinical recommendations to* patients [2]. Considering rapidly accumulating data, the ASRM guidance on this subject also appears incorrect. With at least hundreds of so-far reported perfectly normal births following transfers of selected chromosomal-abnormal embryos [9], one could have expected a more supportive guidance for such embryo transfers, especially since IVF centers have become hesitant about dispositions of “abnormal” embryos in view of the growing controversy on the subject. Consequently, thousands of “abnormal” embryos with significant pregnancy and live birth potential are accumulating in IVF centers around the world, caught in limbo, since centers do not transfer but also do not discard them.

It appears that current diagnostic criteria used by PGT-A laboratories worldwide are not based on validated well designed studies. Unless better and truly validated technologies are developed, PGT-A should not be utilized in routine IVF cycles [1, 5]. We also wish to reemphasize that we do not believe that reputable professional organizations should leave formal guidance for a crucially important add-on to IVF to professional societies with obvious conflicts of interests. Instead, reputable unconflicted professional societies should develop their own clearly understandable and transparent guidance before government organizations start intervening and assume responsibilities for guidance. ACOG and/or ASRM in the U.S., and ESHRE in Europe should lead the way without undue influence.

## Data Availability

N/A

## Abbreviations

ACOG: American College of Obstetricians and Gynecologists
ASRM: American Society for Reproductive Medicine
CoGEN: Controversies in Preconception, Preimplantation and Prenatal Genetic Diagnosis
DNA: Deoxyribonucleic acid
ESHRE: European Society for Human Reproduction and Embryology
GCPG: Genetic Counseling Professional Group (of the ASRM)
IDNHG-IVF: International Do No Harm Group in IVF
NGS: Next Generation Sequencing
PGDIS: Preimplantation Genetic Diagnosis International Society
PGT-A: Preimplantation genetic testing for aneuploidy
